# Human activity recognition using wearable sensors, discriminant analysis, and long short-term memory-based neural structured learning

**DOI:** 10.1038/s41598-021-95947-y

**Published:** 2021-08-12

**Authors:** Md Zia Uddin, Ahmet Soylu

**Affiliations:** 1 SINTEF Digital, Oslo, Norway; 2 Norwegian University of Science and Technology - NTNU, Gjøvik, Norway; 3 OsloMet - Oslo Metropolitan University, Oslo, Norway

**Keywords:** Computational science, Computer science, Information technology

## Abstract

Healthcare using body sensor data has been getting huge research attentions by a wide range of researchers because of its good practical applications such as smart health care systems. For instance, smart wearable sensor-based behavior recognition system can observe elderly people in a smart eldercare environment to improve their lifestyle and can also help them by warning about forthcoming unprecedented events such as falls or other health risk, to prolong their independent life. Although there are many ways of using distinguished sensors to observe behavior of people, wearable sensors mostly provide reliable data in this regard to monitor the individual’s functionality and lifestyle. In this paper, we propose a body sensor-based activity modeling and recognition system using time-sequential information-based deep Neural Structured Learning (NSL), a promising deep learning algorithm. First, we obtain data from multiple wearable sensors while the subjects conduct several daily activities. Once the data is collected, the time-sequential information then go through some statistical feature processing. Furthermore, kernel-based discriminant analysis (KDA) is applied to see the better clustering of the features from different activity classes by minimizing inner-class scatterings while maximizing inter-class scatterings of the samples. The robust time-sequential features are then applied with Neural Structured Learning (NSL) based on Long Short-Term Memory (LSTM), for activity modeling. The proposed approach achieved around 99% recall rate on a public dataset. It is also compared to existing different conventional machine learning methods such as typical Deep Belief Network (DBN), Convolutional Neural Network (CNN), and Recurrent Neural Network (RNN) where they yielded the maximum recall rate of 94%. Furthermore, a fast and efficient explainable Artificial Intelligence (XAI) algorithm, Local Interpretable Model-Agnostic Explanations (LIME) is used to explain and check the machine learning decisions. The robust activity recognition system can be adopted for understanding peoples' behavior in their daily life in different environments such as homes, clinics, and offices.

## Introduction

Body sensors have been getting popular day by day for various practical applications such as entertainment, security, and healthcare research fields. The wearable sensors can be actively explored applied for accurately recognizing people’s health status, activities, and behavior. Thus, these sensors can be promising to improve our life much in the same way as other regular electronic devices such as the personal computers, smart phones, etc. In case of commercial applications, wearable sensors have been mostly applied to trigger panic buttons to seek emergency whenever necessary. Such use of the sensors can be considered as a commercial success ^1^. In such cases, the users are guessed to be alert and fit enough to use the button. Also, the panic button should be designed as light and comfortable to wear. Wearable sensors have also attracted many researchers of medical sciences to observe physiological behavior of human body. In such applications, patients’ vital body signs are continuously observed such as heart rate, respiration, etc. ^2^.

Alongside other applications of the body sensors, they can be used to obtain necessary treatment at home, especially for chronic patients of heart-attacks, Parkinson disease, etc. For example, patients usually go under the rehabilitation process after an operation where they should follow strict daily routines. In such cases, wearable sensor-based systems can help to monitor the health status and behaviors of the patient using the physiological signals. During the rehabilitation stage, the wearable sensors can also provide audio feedback or other rehabilitative services as well. The history of the patient’s health and behaviors can be monitored remotely by doctors, relatives, or caregivers etc.

For observing behavior based on wearable sensors, research is still going on these days towards developing smart healthcare systems such as fall detection of elderly living alone at home^4–6^. Also, wearable devices and sensors have been getting attentions for commercial purpose such as smartwatch and Google’s smart glasses. Thus, the wearable technologies can have an important impact in medical technologies to define doctor-patient relationship and saving healthcare cost. The rapid growth of the applications of wearable technologies, their acceptance seems to continue in many important sectors such as healthcare.

Wearable sensors-based activity recognition system handles the integration of sensing and reasoning to be able to better understand people's behavior ^7–9^. Research in human behavior analysis has become popular in many areas (e.g., surveillance, context-aware systems, and ambient assistive living). In ^7^, the authors focused on important applications of activity recognition in several fields such as healthcare, wellbeing and sports systems. Regarding the wearable sensor-based activity recognition, they reported examples of healthcare monitoring and diagnosis systems; rehabilitation; child and elderly care. Besides, they also reviewed monitoring systems to improve the life and ensure safety and well-being of children, seniors, and people who have cognitive disorders. In ^8^, the researchers proposed activity recognition systems as links among the common diseases with the degrees of peoples' physical activity ^8^. The authors also analyzed the systems with daily activity patterns that contributed well to the procedure and diagnosis of neurological disorders. In ^9^, the authors proposed an activity prediction approach based on sensors embedded in smartphones to estimate energy expenditure recognizing spontaneous physical activities.

**Figure 1:**
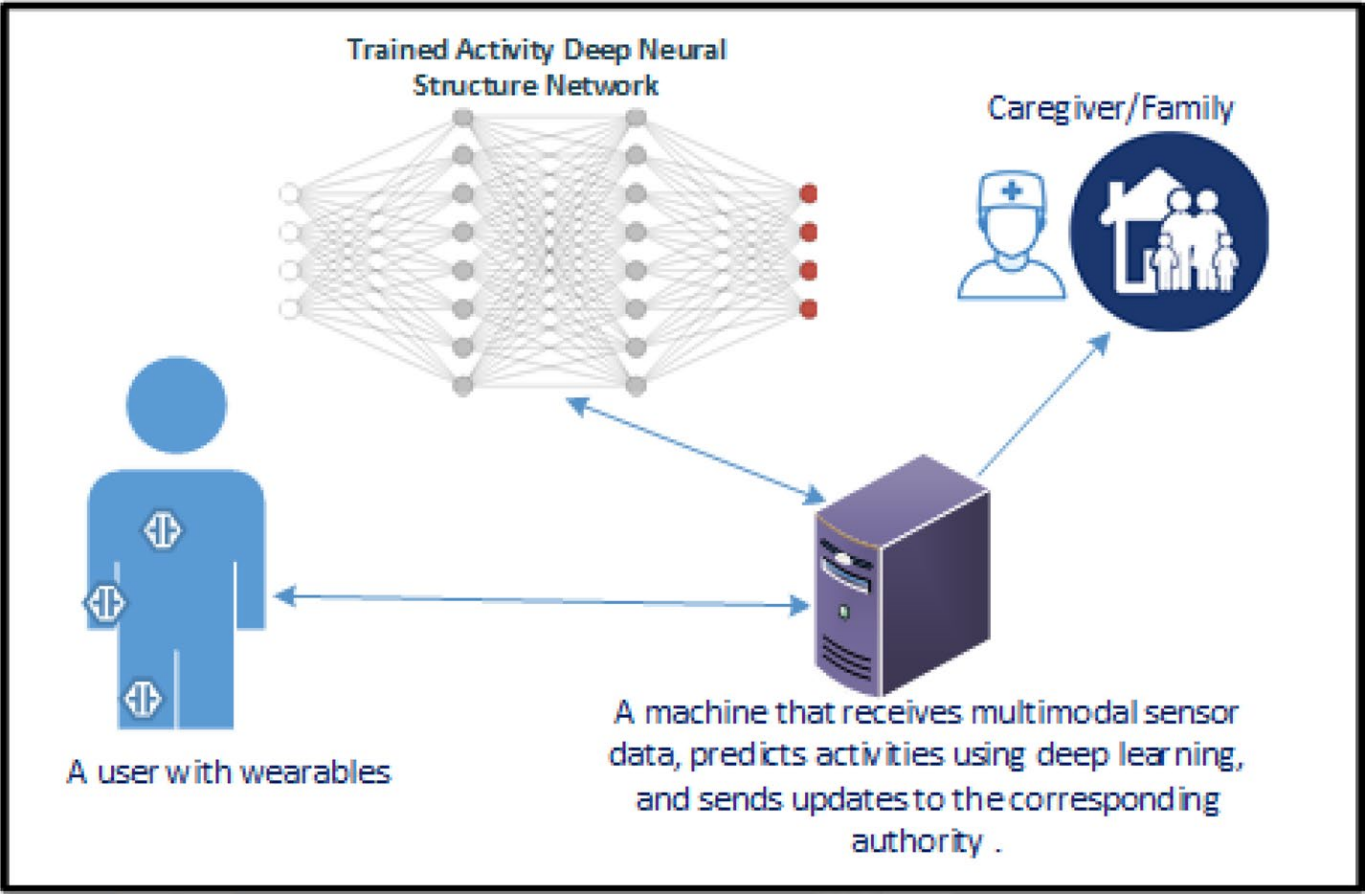
A schematic setup for wearable sensor-based human activity recognition system.

Sensor-driven systems are usually based on the collaboration between the users and technology where the system is aimed to provide a good support in decision support systems ^10^. So, there should always be enough balance between the rights of the users and requirements of an efficient functioning of the system. Among the other sensors as data sources for human behavior analysis, cameras are the very popular since the users are visually available in the display ^11,12^. Though they are very popular, such systems can however raise privacy issues of the users often. On the other side, wearable sensors sensor-based approaches for activity recognition system do not usually face privacy issues ^13^]. Figure 1 shows a schematic setup of a body sensor-based human activity recognition system where a user is wearing some sensors in different body parts such as chest, wrist, and ankle. The multimodal sensor data is transferred to a computer though wireless medium and then the data is processed there to perceive underlying events via deep learning.

The recent success of machine learning models has been mostly possible due to efficient deep learning algorithms with hundreds of layers and millions of parameters ^14–22^. Among the successful deep learning algorithms, Deep Belief Network (DBN) was the first successful machine learning technique which was later overpowered by Convolutional Neural Networks (CNN), especially for image processing and computer vision applications. ^22^. CNN-based deep learning is very robust for recognizing patterns in images, but it has not been much applied for time-sequential data. In that regard, Recurrent Neural Networks (RNNs) have been quite popular to model time-sequential events in data ^23–28^. However, general RNN consists of a problem called vanishing gradient limitation that basically occurs in processing of long-term information. To overcome that, Long Short-Term Memory (LSTM) was developed consisted of different memory units were included ^27^. Though the aforementioned deep learning approaches are good in corresponding fields, they are still sensitive to noise hence noise in testing data may drop the accuracy of the overall performance of the systems.

Google's open-source tool TensorFlow is one of the most famous tools applied among the deep learning tools for different event modelling such as prediction related tasks in pattern recognition areas. Neural Structured Learning (NSL) ^29^, an open-source framework inside that is one of the latest deep learning algorithms to learn events in data. NSL can be used to construct robust models in a wide range of research fields such as computer vision and natural language processing. It can be utilized for training taking the advantage of structured signals related to the feature inputs. NSL is a neural graph learning approach to train neural networks depending on graphs and structured data ^30^. NSL also generalizes basic adversarial learning ^31^ utilizing the structured data with good relational information among the samples. The structured signals are applied to update the learning parameters to train a network towards learning as accurate as possible alongside maintaining the structures of the inputs to the network. Hence, NSL seems to be a good choice in this activity recognition work for better activity modeling and testing than the other traditional deep learning approaches such as DBN, CNN, and LSTM-based RNN. Besides, the aforementioned traditional neural networks can be put inside an NSL framework to improve a system's performance. For instance, LSTM is fed into NSL in this work to model time-sequential wearable sensor data for activity modeling.

In the recent time, Artificial Intelligence (AI) has reached in an extraordinary momentum. Proper control and exploration can bring the best of expectations of it in many practical application fields. Towards meeting the expectations as fast as possible, the corresponding community is now facing the barrier of explainability problem to open the black-box machine learning models, which is called explainable AI (i.e., XAI). XAI is a key feature for the applications of AI models and seems to continue its trend in future as one of the core research focuses of the AI research community. Among the popular state-of-the-art algorithms for XAI (e.g., Local Interpretable Model-Agnostic Explanations (LIME) and SHapley Additive exPlanations (SHAP)), LIME is light-wight to generate quick and satisfactory post-hoc explanations ^32–35^. Therefore, LIME seems to be a suitable choice for this work to apply XAI on the decision provided by the machine learning model.

In this work, a novel activity recognition method is proposed based on applying NSL on the wearable sensor data. At first, robust statistical features are extracted from the sensors followed by applying kernel discriminant analysis (KDA) to make them more robust. Then, NSL consists of LSTM inside, is applied to model the features for activity training and recognition. The proposed method based on NSL with LSTM inside, should yield better recognition performance than the traditional approaches such as DBN, CNN, and RNN. Since the approach is fast and efficient, it can be tried on various smart environments such as smart homes or clinics. Furthermore, LIME is used for post-hoc explanations of the machine learning decision of activities.

## Methodology

**Figure 2:**
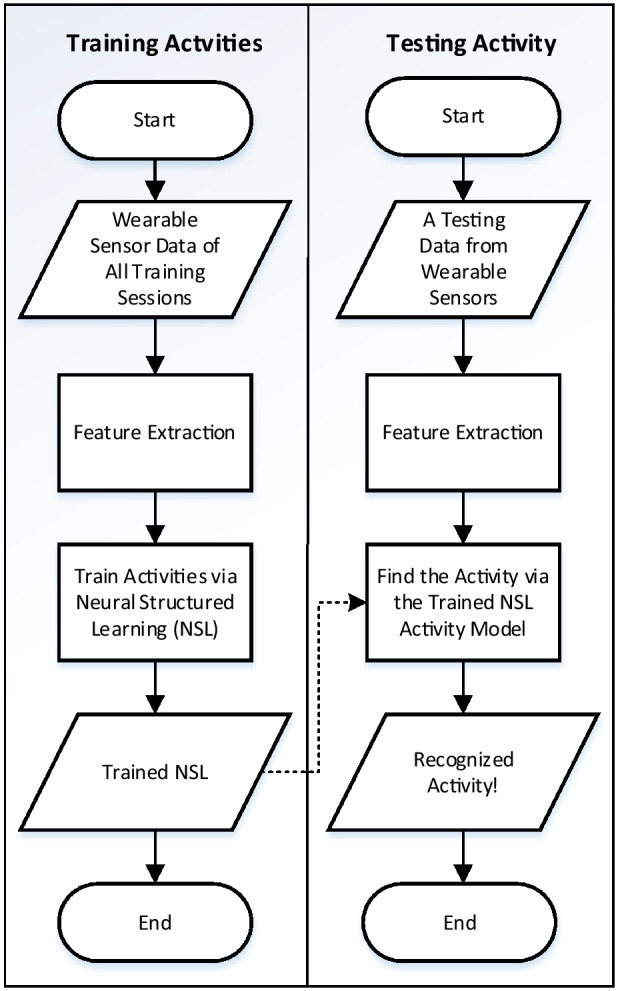
Flowchart of training and testing process of the proposed method.

The system starts with the processing of data obtained from different wearable sensors and take the decision regarding the underlying activities via feature processing and training a robust activity model using NSL. Figure 2 shows the general flowcharts for the training and testing of the proposed method.

### Sensor data processing

In this work, a public dataset called MHEALTH ^36,37^ was majorly used to check the performances of different approaches where data was collected from different sensors worn on the body of different subjects. The dataset comprises vital signs and motion recordings of ten subjects of the diverse profile. In dataset, there were twelve activities performed by the subjects and they are: Standing still (A1), Sitting and relaxing (A2), Lying down (A3), Walking (A4), Climbing stairs (A5), Waist bends forward (A6), Frontal elevation of arms (A7), Knees bending (A8), Cycling (A9), Jogging (A10), Running (A11), and Jump front and back (A12).

**Figure 3:**
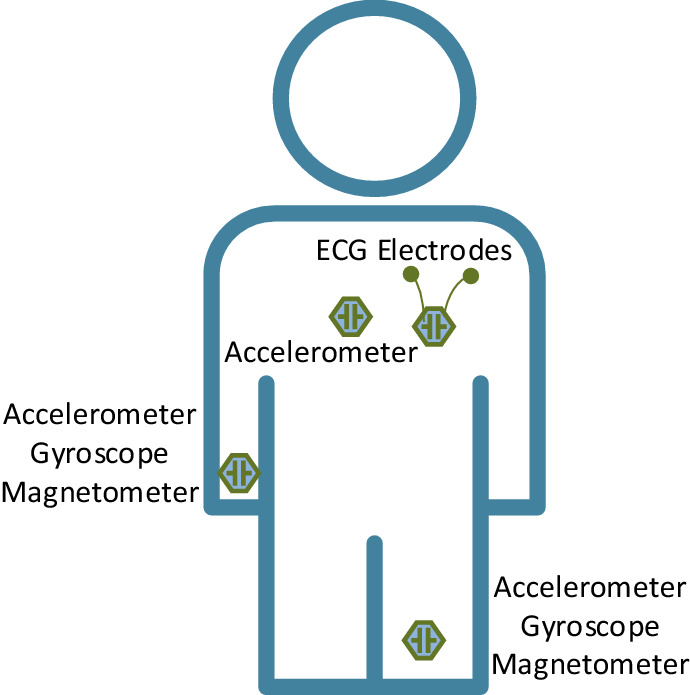
Sensor placements for MHEALTH dataset.

During building the dataset, the sensors were placed in different places of the body of the subjects (i.e., chest, right wrist, and left ankle) and the data was collected with the sampling rate of 50 Hz. The multimodal recording of the data allows to record different important data such as data from the heart via ECG sensors, body acceleration via accelerometers, tilting amount via gyroscopes, and magnetic field orientation of the body via magnetometers. Figure 3 shows the sensor placements on subjects' body for the MHEALTH dataset.

The sensors are worn on different body parts i.e., the heartrate healthcare sensor on the chest provides heart data measurement and other sensors provide the motion-related experience of different parts of the body e.g., the acceleration by accelerometers, the rate of tilting by gyroscopes, and the magnetic field dynamics by the magnetometer. Shimmer2 sensors were used for the data collection ^38^. The utilization of multiple sensors allows the work to calculate the motion observed by different body parts such as the acceleration, turning rate, and magnetic field orientation. Thus, it helps better perception of the users' body dynamics.

### Feature representation

The sensor data is organized to represent features as follows. The acceleration data from the chest sensor is represented as

1 $${C}_{C}=({C}_{x},{C}_{y},{C}_{z}).$$

Electrocardiogram (ECG) data from the heartrate sensor lead 1 and 2 are obtained as

2 $$E={(E}_{1},{E}_{2}).$$

Acceleration from the left-ankle is obtained as

3 $${C}_{LA}=({L}_{x},{L}_{y},{L}_{z}).$$

Tilting data from the left-ankle Gyroscope sensor is obtained as

4 $${Y}_{LA}=({L}_{x},L,{L}_{z}).$$

Left-ankle magnetometer data is obtained as

5 $${M}_{LA}=({G}_{x},{G}_{y},{G}_{z}).$$

The left-wrist accelerometer data is obtained as

6 $${C}_{RW}=({I}_{x},{I}_{y},{I}_{z}).$$

Titling data from the left-wrist gyroscope is obtained as

7 $${Y}_{LW}=({R}_{x},{R}_{y},{R}_{z}).$$

Gyroscope sensor data from the right-wrist is obtained as

8 $${Y}_{RW}=({Q}_{x},{Q}_{y},{Q}_{z}).$$

Right-wrist magnetometer sensor data is obtained as

9 $${M}_{RW}=({T}_{x},{T}_{y},{T}_{z}).$$

Furthermore, all the data features obtained for a specific time-period are augmented and represented as

10 $$L={C}_{C}||E||{C}_{LA}||{Y}_{LA}||{M}_{LA}||{C}_{RW}||{Y}_{LW}||{Y}_{RW}||{M}_{RW}.$$

**Figure 4:**
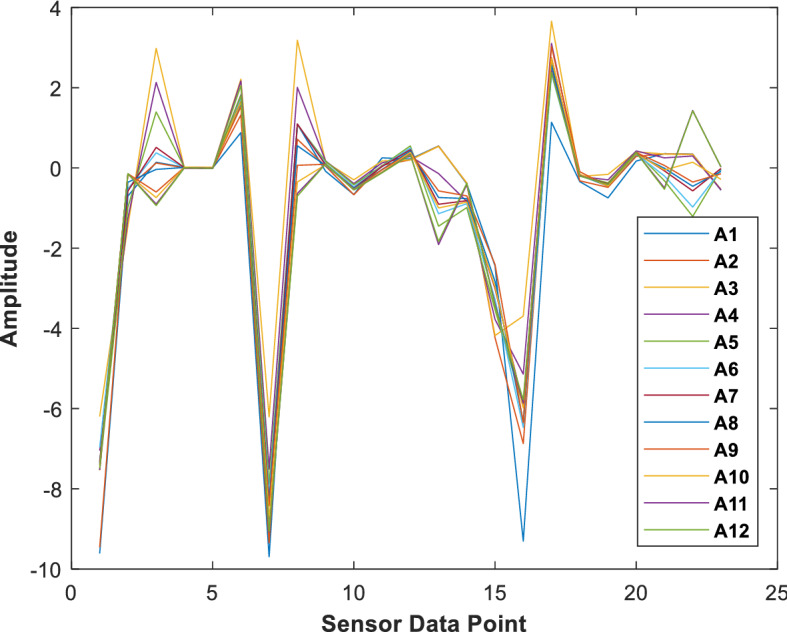
Mean of the 23 raw data point (i.e., *L*) from twelve different activities.

Figure 4 shows the mean *L* features of twelve different activities where it can be noticed that different activities follow different structures. That indicates that a robust classification system can make them separated from each other. The total number of samples is 343,070 × 23 for all activities of 10 subjects. The samples are reshaped as 34,307 × 10 × 23 i.e., per second. Also, in the dataset, to define the gap (interval) in between the activities, the dataset authors included null or 0 classes. Besides, Table 1 shows the features from the individual sensors on body.

**Table 1:** Features from MHEALTH dataset.

Features
Chest accelerometer *C*_*c*_
ECG sensor data on chest *E*
Left-ankle accelerometer *C*_*LA*_
Left-ankle gyroscope *Y*_*LA*_
Left-ankle magnetometer *M*_*LA*_
Left-wrist accelerometer *C*_*RW*_
Left-wrist gyroscope *Y*_*LW*_
Right-wrist gyroscope *Y*_*RW*_

**Figure 5:**
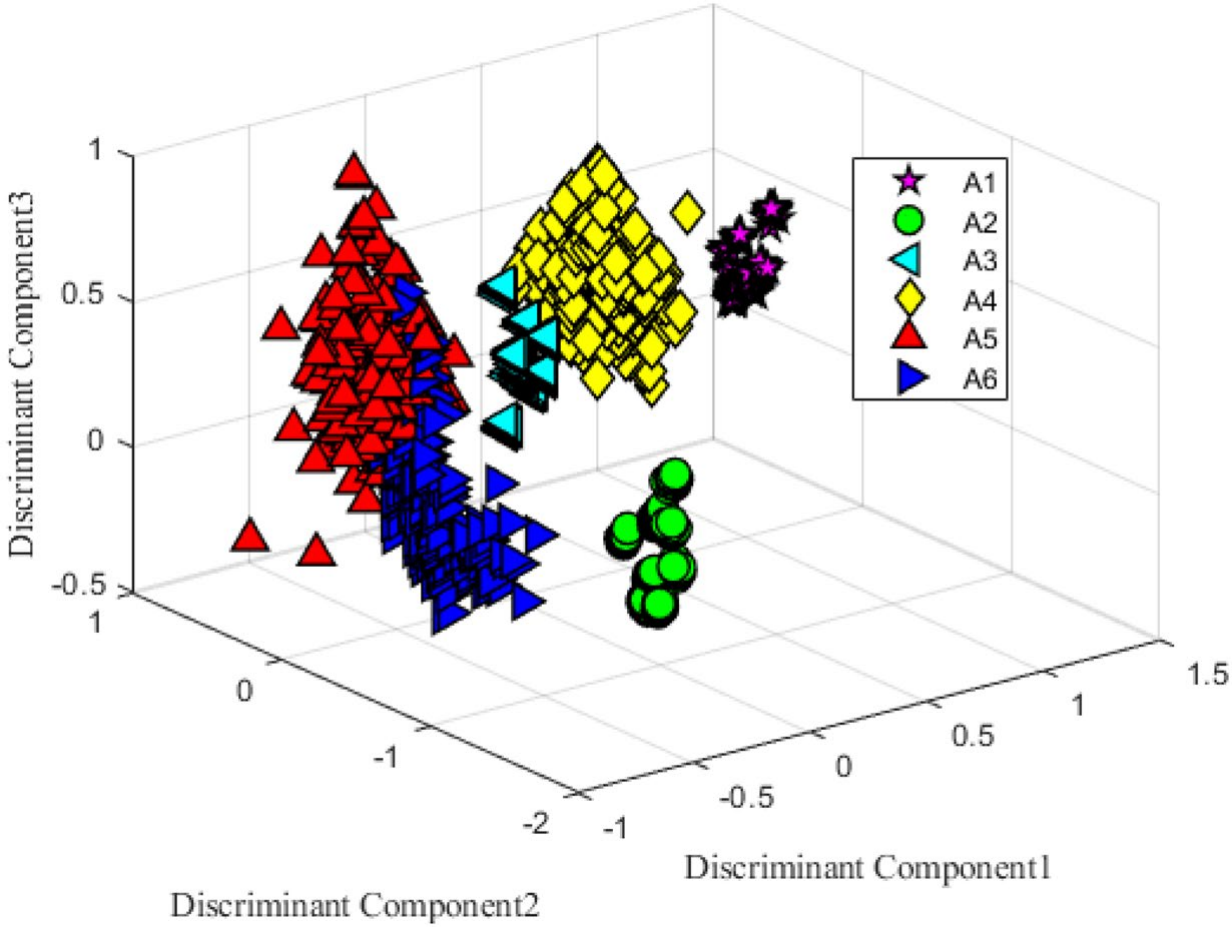
Discriminant analysis projection plot of the samples from six activities.

To enhance the features, kernel-based discriminant analysis (KDA) is applied. KDA is based on an eigenvalue resolution problem based on a kernel to find a nonlinear space that tries to minimize the inner-class scatterings of the samples from different classes while maximizing the inter-class scatterings. In this work, features from different body sensors' data goes through a Gaussian kernel to find out the nonlinear feature space, which is obtained from the maximization of the following as

11 $$D=\frac{|{D}^{T}{C}_{W}D|}{|{D}^{T}{C}_{B}{D}|},$$

where *D* represents discriminant features of between-class scatterings ${C}_{B}$ and within-class scatterings ${C}_{W}$. The eigenvalue problem

12 $${C}_{B}{D}=\Lambda {C}_{W}{D},$$

where $\Lambda$ represents the eigenvalue matrix. Figure 5 shows a 3-D plot of KDA features of the samples from six different classes where it shows a good clustering of the samples.

### Human activity modelling

Proactive machine learning systems require computational models to perceive and anticipate upcoming unknown future events. These models very often need to have an internal model that can learn to structure the temporal phenomena. Deep artificial neural networks (ANNs) is mostly used in this regard to build significantly enhanced predictive technologies. ANN models are designed with various architectures that would be appropriate for specific machine learning task consists of specific computational models. Among which, recurrent neural networks (RNN) is one that can learn from sequences of data and convolutional neural networks (CNNs) with a capability of learning from mostly image type of data. So, LSTM seems appropriate over CNN due to its time-sequence modelling capability.

LSTM RNNs are basically applied to sequences of information in a one-at-a-time way i.e., the model predicts mostly the very next element of the sequence. So, a little variation in the input data (e.g., noise) may cause wrong perceptions by the model. Hence, this work focuses on the variations of temporality in a time-sequential data-based system that should allow us to a come up with a better activity model. That is why, the proposed approach goes for LSTM inside Neural Structured Learning (NSL) for robust activity modelling.

Neural Structured Learning (NSL) is a machine learning approach that is targeted on training the neural networks by taking the advantages of structured signals combined with features from the inputs ^25^. In NSL, the structured signals are accustomed to regularizing working out of a neural network that has the strong target to understand accurate predictions with the aid of minimizing supervised loss. Simultaneously, it tries to steadfastly keep up structural similarity of the input by emphasizing the minimization of the neighbour. The generalized neighbour loss equation could be represented as bellow.

13 $$neighbour\_loss ={\sum }_{k=0}^{W}L({y}_{i, }{\widehat{y}}_{i})+\propto {\sum }_{k=0}^{W}L\left({y}_{i, }{x}_{i,}N\left({x}_{i}\right)\right).$$

**Figure 6:**
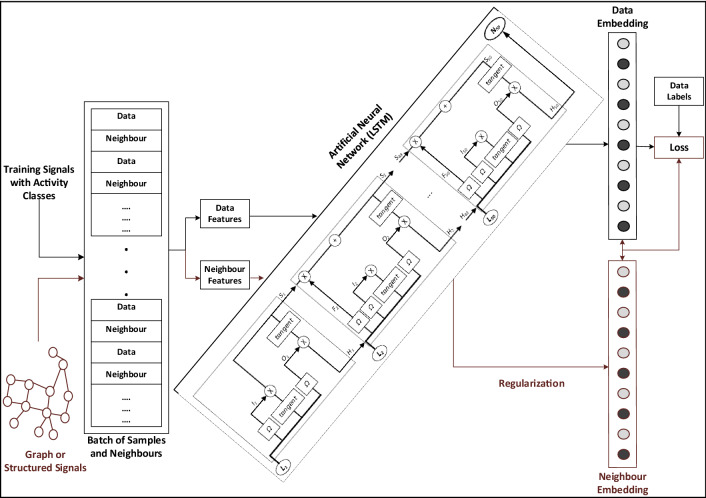
An LSTM-based neural structured learning model for human activities.

Thus, NSL basically generalizes the network using two different ways. The first one is by using neural graph learning where neighbors are connected by a graph. The second one is by utilizing adversarial learning where the neighbors are induced by the adversarial perturbation ^30^. The overall workflow of an LSTM-based NSL model for human activities, is depicted in Fig. 6. In the figure, the black arrows show the flow how training is done and in the same figure, the red arrows depict how the learning takes the advantage of structured signals. In NSL, the training data is represented by augmentation of the structured signals. When the structured signals are not possible to obtain, in that case the signals are usually constructed by process of adversarial learning. Once the training samples are augmented, which consists of original and neighbouring samples, they are applied on an LSTM neural network first that consists of several memory units to calculate the samples' embeddings. In the LSTM depicted in the Fig. 6, L is the input to the LSTM sequential units and N is the final output. Then, the neighbour loss is calculated by finding the distance between the embedding of a sample and neighbour itself, i.e., regularization which is later added to the final loss. While regularizing the neighbour-based process, the layers in the neural network of NSL can be used to calculate the loss. For adversarial-based (i.e., induced) regularization, the neighbour loss is computed based on the distance between the ground truth and predicted output of the adversarial neighbors. Table 2 shows the NSL model summary consisting of layers and parameters used in this work.

**Table 2:** The NSL models used in this work.

Layer	Output shape	Number of parameters
LSTM	50	14,800
Dense-1	128	6528
Dense 2	12	1548
Total parameters	22,876	

## Results and discussion

**Figure 7:**
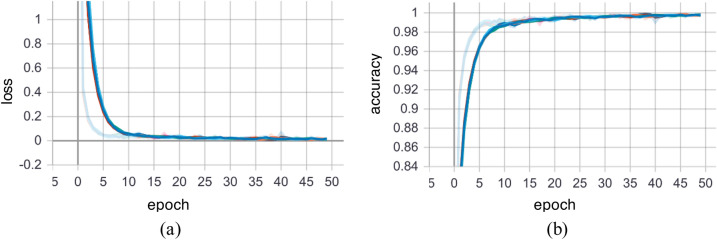
**(a)** Loss and **(b)** accuracy of the NSL model for 50 epochs and 10 folds.

**Figure 8:**
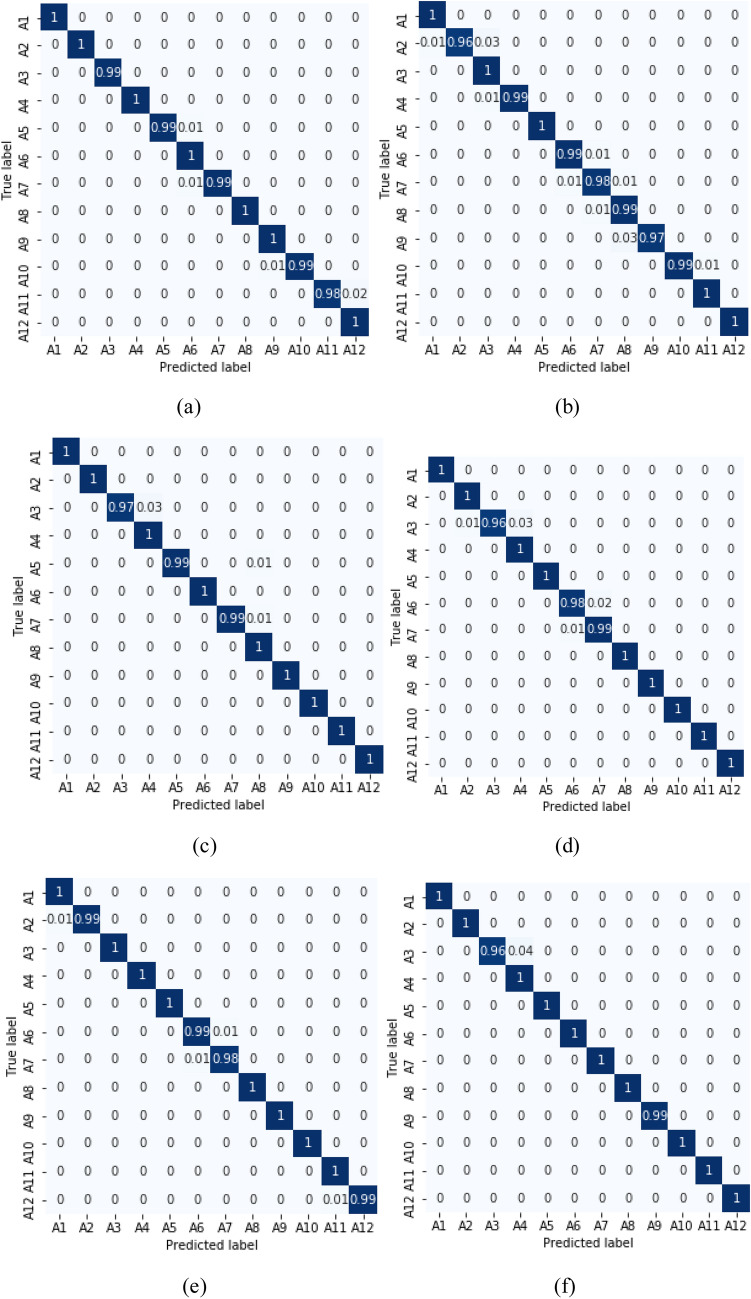
Confusion matrices of fold 1–6 from **(a)** to **(f)** using NSL.

**Figure 9:**
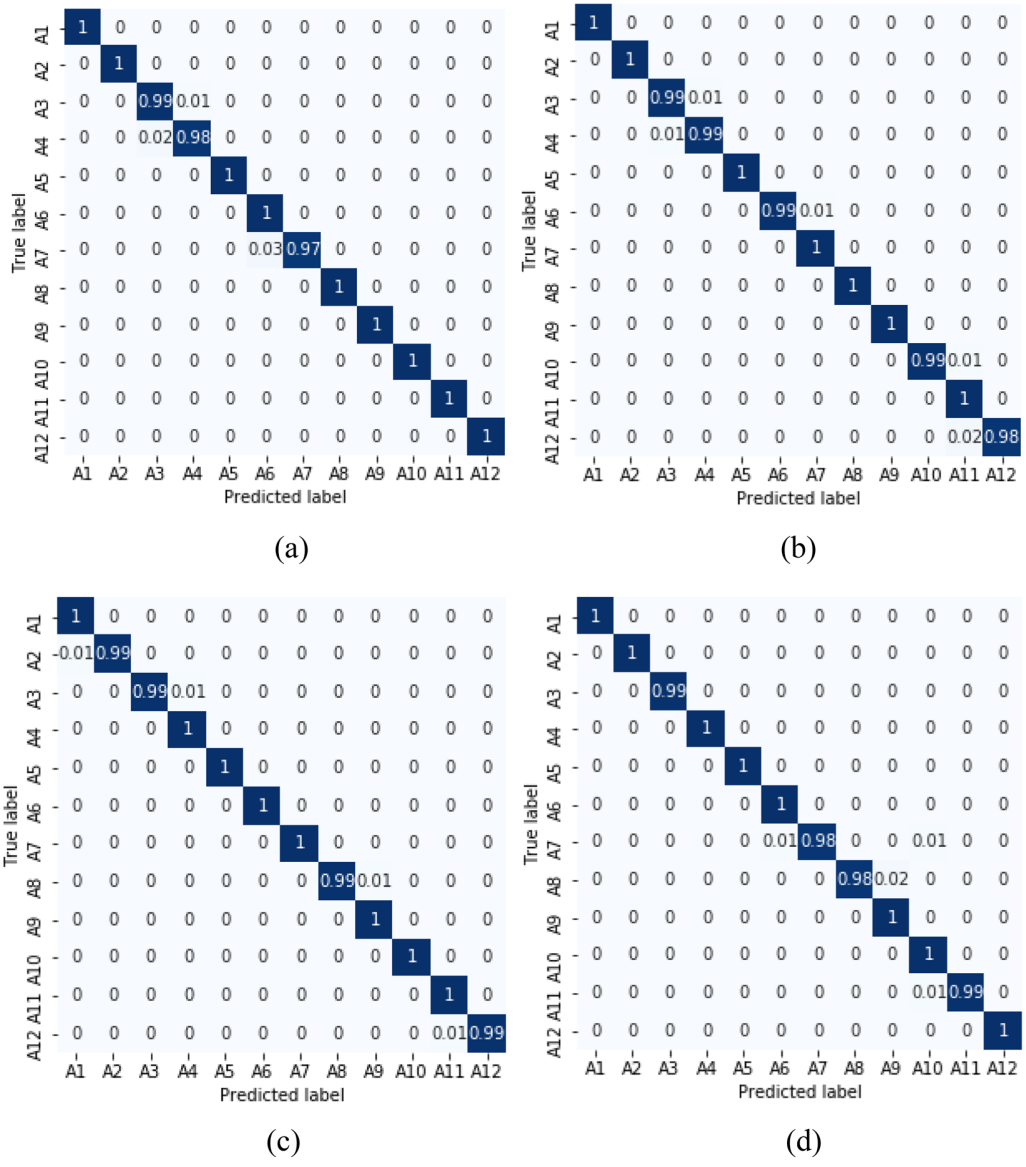
Confusion matrices of fold 7–10 from **(g)** to **(j)** using NSL.

**Figure 10:**
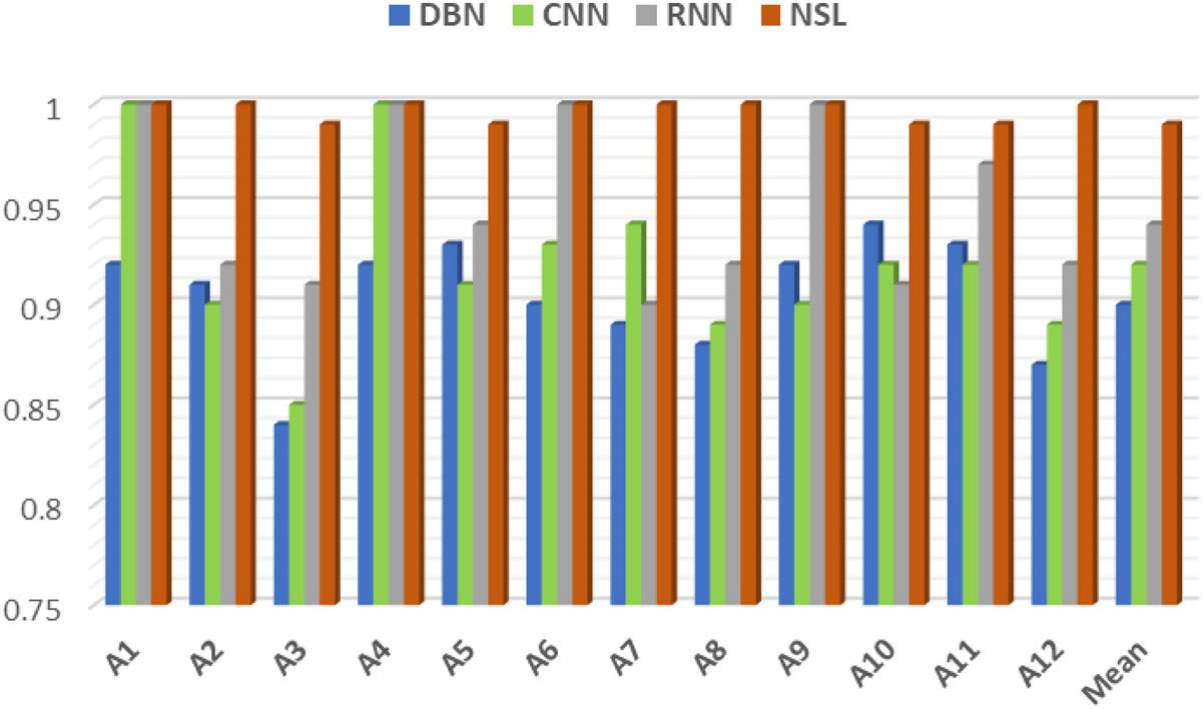
Mean of the recalls of the activities using four different approaches.

Ten-fold cross validations (i.e., leave-one-subject-out) is applied for the experiments of twelve activities performed by ten subjects. The leave-one-subject-out validation basically repeatedly splits the dataset according to the number of subjects. One subject is selected at a time for the testing purposes while the others are used for training the machine learning model. This process is repeated until all the subjects have been used as testing part. Finally, overall accuracy is calculated based on all the folds of the subjects to show the performance of the approach. The proposed NSL-based approach that achieved the mean recognition performance of 99% as shown in Table 3. Figure 7 shows the loss and accuracy of the ten-fold LSTM-based NSL model applied in this work. The confusion matrices of the folds from the ten-fold cross-validation are reported in Figs. 8 and 9. To compare the proposed approach with other traditional approaches such as typical DBN, CNN, and RNN-based experiments ^39^ were done. However, they achieved a maximum mean recognition rate of 94%. Figure 10 also depicts that the NSL-based proposed approach overpowers three other state-of-the-art approaches. The applied training and testing NSL method is quite fast, can be applied real-time. To avoid the overfitting problem, minimum number of epochs were considered that achieved the constant accuracy of on the testing results.

**Table 3:** The mean recall rates of different activities using different approaches.

Activity/Model	DBN	CNN	RNN	NSL
Standing still (A1)	0.92	1.00	1.00	1.00
Sitting and relaxing (A2)	0.91	0.90	0.92	1.00
Lying down (A3)	0.84	0.85	0.91	0.99
Walking (A4)	0.92	1.00	1.00	1.00
Climbing stairs (A5)	0.93	0.91	0.94	0.99
Waist bends forward (A6)	0.90	0.93	1.00	1.00
Frontal elevation of arms (A7)	0.89	0.94	0.90	1.00
Knees bending (A8)	0.88	0.89	0.92	1.00
Cycling (A9)	0.92	0.90	1.00	1.00
Jogging (A10)	0.94	0.92	0.91	0.99
Running (A11)	0.93	0.92	0.97	0.99
Jump front and back (A12)	0.87	0.89	0.92	1.00
Mean	**0.90**	**0.92**	**0.94**	0.99

Local explanations in machine learning models handle explainability by dividing the model's complex solutions space into several less complex solution subspaces that are relevant for the model. These explanations can adopt some techniques with the differentiating property to interpret the model to some extent. Local explanations are one within that category as simplified machine learning models are sometimes just representative of some specific sections of a model. Most of the methods of model simplification are basically based on rule extraction techniques. However, the most popular contributions for local explanation are based on the approach called Local Interpretable Model-Agnostic Explanations (LIME) ^32–35^. LIME usually generates locally linear models for the predictions of a machine learning model to explain it. Explanations by simplification in LIME builds a whole new system based on the trained model to be explained. Then, the new simplified model usually tries to optimize its resemblance to its predecessor model functions while reducing the complexity and keeping a similar performance at the same time.

**Figure 11:**
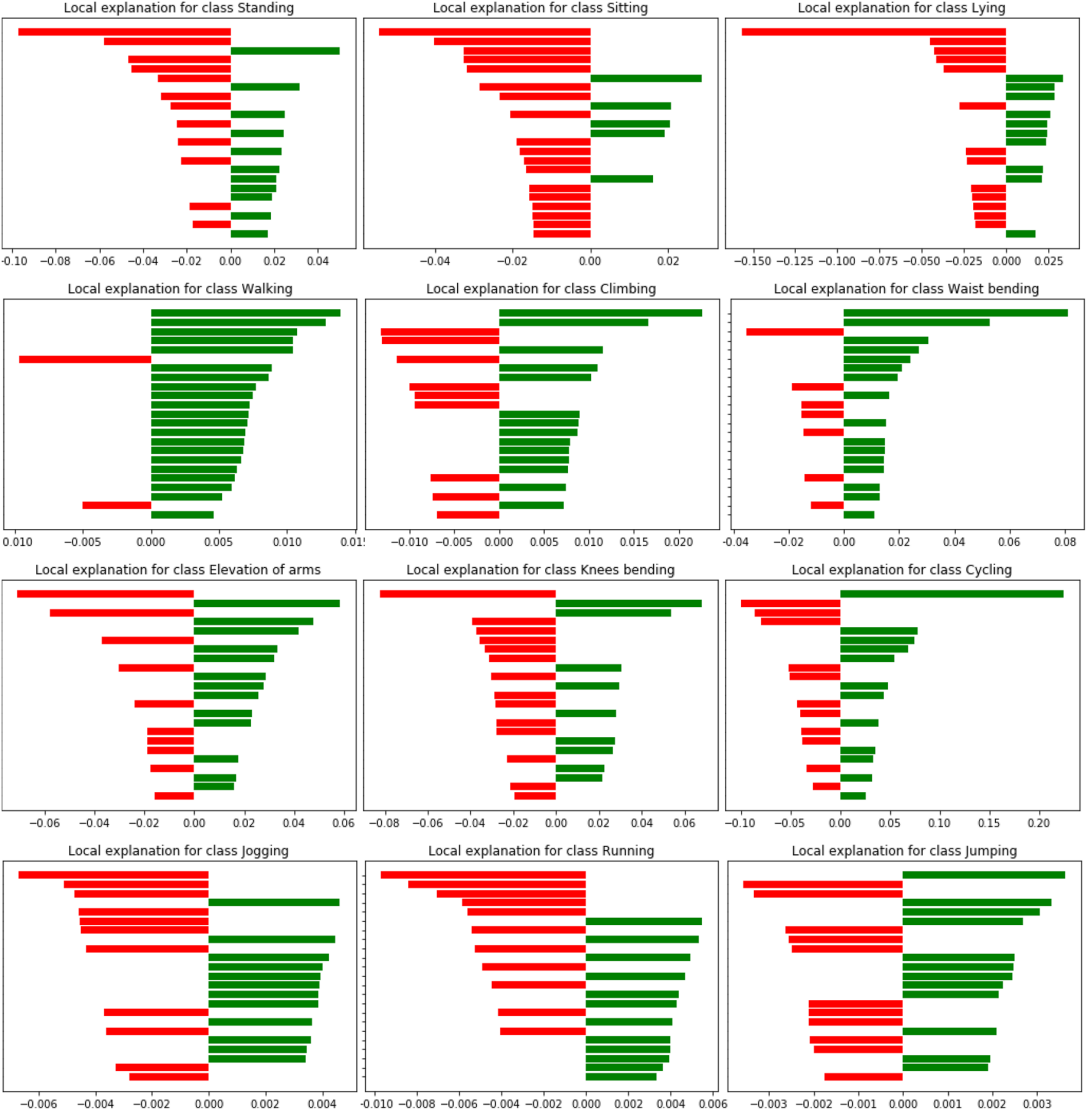
Explanation results using LIME on a test sample of walking activity.

**Figure 12:**
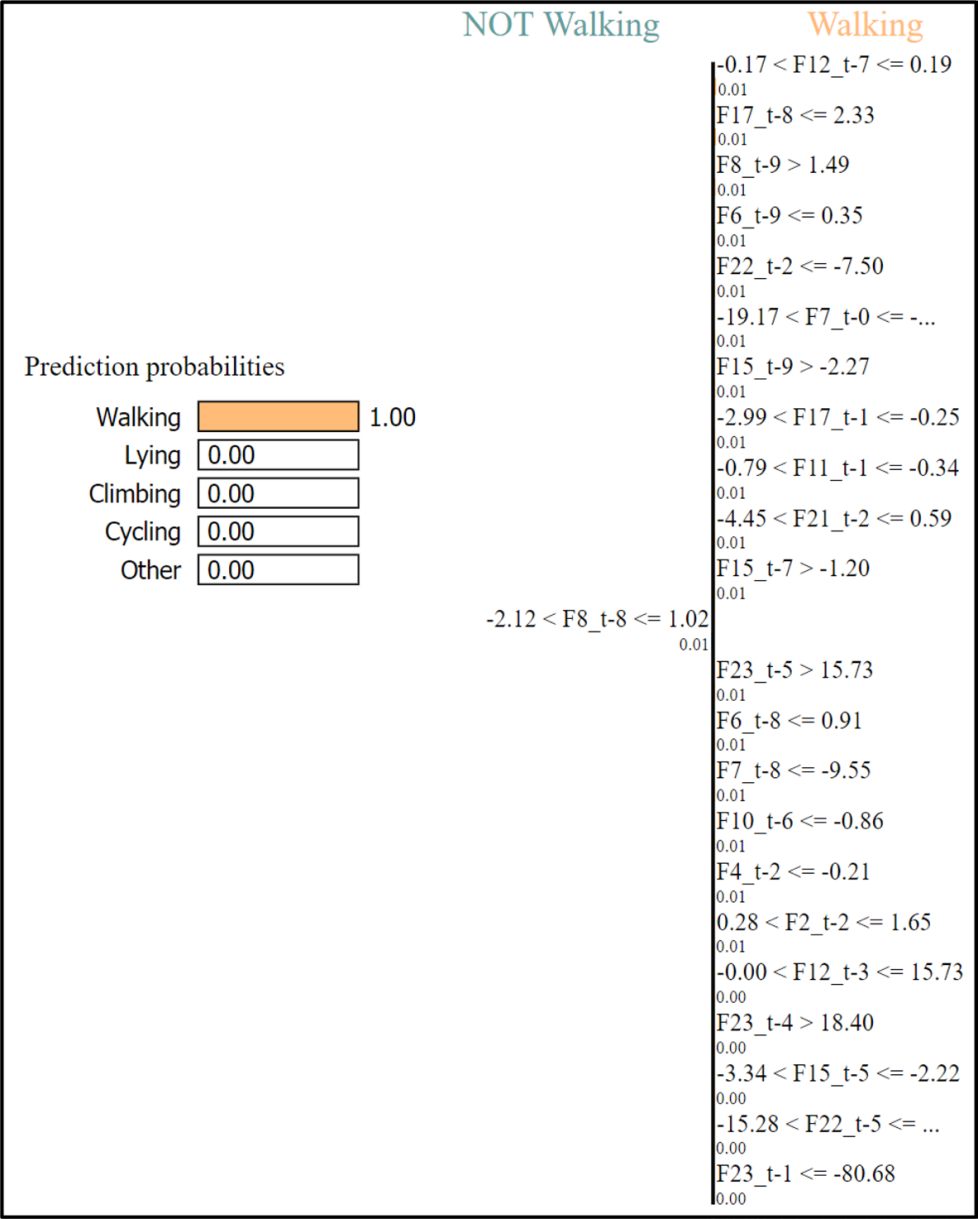
Prediction probabilities of the activities and feature range as well as probabilities of walking versus others, for a test sample of walking activity.

Thus, we tried LIME in this work to explain our trained model. Figure 11 shows the LIME explanation on the LSTM-based trained model for a sample walking test sample from the dataset. In each subfigure om the figure, the right side (i.e., green bars) represents the weights for that activity and other side (i.e., red bars) for other activities. The bars from top to bottom in the subfigures represent the features 1 to 23, respectively. As can be noticed there, the collective weights of walking activity are quite larger than that of the other activities, compared to similar local explanations in case of other activities. This indicates that, the test sample belongs to walking activity that also matches the ground truth as well as model predictions for the sample. Figure 12 also shows further explanations consisting of the prediction probabilities of the activities and feature range as well as probabilities of walking versus other activities, for a test sample from walking activity. Thus, the Figs. 11 and 12 justify and explain the sample to be in the walking activity.

Though collecting data and sharing it most of the time improves the services provided to the users, it may however increase the risk of the data protection right of the users. Hence, data is recommended to be preserved well. So, it should be logical to point out that on one side, technology may bring threats to the user's personal rights regarding privacy and data protection. Nevertheless, technologies can be adopted to solve the problems they create while enhancing their uses in compliance with the requirements of privacy, by choosing the data sources as much anonymous as possible if required such as body sensor data over cameras for monitoring of the users. Most of the smart services provided in a smart environment such as smart homes/ clinics include observing residents’ or users' activities to analyse patterns of their daily activities to improve their health and lifestyles. Some services focus one detecting emergency (e.g., falls or heart attacks) which needs urgent medical attention. Though a lot of research has been done in this regard, a significant amount of research is still needed to develop robust algorithms for such activity pattern analysis where there would as less false alarms as possible. Wearable sensors basically provide reliable data for activity recognition. However, the main drawbacks of such sensors are intrusiveness and the necessity of frequent recharging of batteries. Besides, wearing such sensors may not be possible always such as by the elderly or the people with memory problems. Though cameras do not generate such drawbacks, they may generate a high risk of privacy since the data is quite visually interpretable most of the time. Thus, wearable sensors seem to be a good choice to generate robust activity recognition model to observe the users' behaviour.

Humans are usually restrained to accept methods that are not interpretable i.e., trustworthy, pushes the demand for ethical machine learning to increase ^40–49^. Focusing only on performance of the models rather than explaining how the decision is taken, gradually pushes the systems towards unacceptance. Though there is a trade-off between the performance and interpretability in machine learning, improvements via explainability can however lead to the correction of the models' deficiencies. Therefore, the machine learning research should focus on generating more explainable models while upholding the high level of accuracies. Lots of research is happening these days on the explainability of machine learning models alongside generating the highly accurate models. Hence, the target here is to focus on extending our activity recognition research to generate a transparent model.

### Results on PUC-Rio dataset

**Figure 13:**
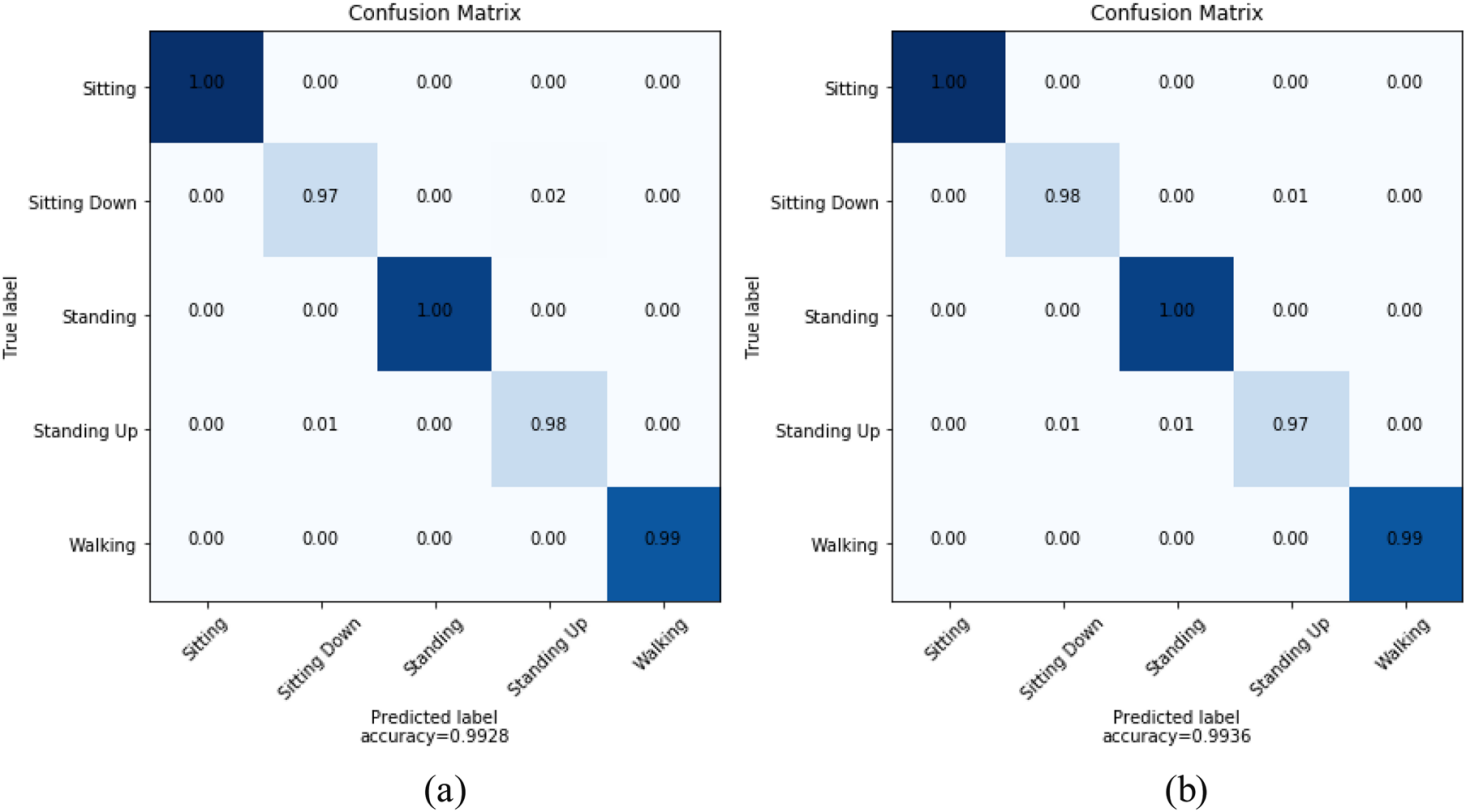
NSL-model performance on **(a)** fold-1 and **(b)** fold-2 of PUC-Rio dataset.

**Figure 14:**
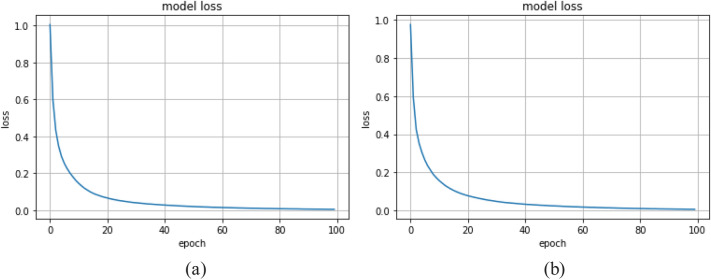
NSL-model error on **(a)** fold-1 and **(b)** fold-2 of PUC-Rio dataset for 100 epochs.

To check the robustness of the proposed method, we applied it on a second dataset named PUC-Rio behavior recognition dataset ^50^. During recording the dataset, a total of four accelerometer sensors were placed in four body positions i.e., left thigh, waist, right ankle, and right arm. The sensors were calibrated before actual data recording. To calibrate the sensors in a standard way, they were placed on a flat table in the same spot. Then, the data collection was done once the sensors were placed on the bodies of the subjects. The database has five activities: sitting, sitting down, standing, standing up and walking. A total of 165,632 different samples are available from the five activities and a two-fold cross validation was applied for the experiments on them. Figure 13 shows the normalized confusion matrices for the two different folds where average accuracy is 99%. However, the traditional approaches such as DBN, CNN could not yield more than 93%. Figure 14 shows error plot during the epochs for training the models for the two folds. Thus, the proposed approach shows its robustness by good performance on the PUC-Rio dataset as well.

## Conclusions

In this work, a multimodal robust human activity recognition system has been investigated using wearable body sensors and robust deep learning method, NSL based on time-sequential data model LSTM inside it. The body sensor data has been analyzed and extracted efficient features based on nonlinear generalized discriminant analysis. The features have been applied to train a deep activity NSL to model twelve different human activities. Finally, the trained model has been applied for recognizing the underlying activity in testing sensor data. Using the proposed approach, maximum mean recall rate of 0.99 has been achieved on MHEALTH activity dataset whereas, the traditional approaches yielded maximum 0.94. The proposed approach also showed 99% accuracy on PUC-Rio behavior recognition dataset whereas, the traditional approaches could not cross 93%. Thus, the experimental results indicated the robustness of the proposed approach. Besides, the fast XAI algorithm LIME has also been tried to justify the decision taken by the machine learning model. The NSL-based multimodal system can be applied or adopted in any health care service for real-time recognition of human activity recognition for better life care of the users, especially the elderly or disabled people to improve their lifestyle and prolong their independent living. While the overall system of monitoring peoples' behavior is important and at the same time technically challenging. There is also an issue of privacy where the users may appreciate the increase safety and data privacy that smart monitoring system can provide. Also, the users may hesitate to be monitored always by any kind of sensors, non-camera-based approaches are acceptable to some extent though. Thus, one of the key challenges here is to determine an acceptable trade-off between privacy intrusion and efficient system.

## References

[1] Edwards J (2012). Wireless sensors relay medical insight to patients and caregivers [special reports]. IEEE Signal Process. Mag..

[2] Malhi K, Mukhopadhyay SC, Schnepper J, Haefke M, Ewald H (2012). A Zigbee-based wearable physiological parameters monitoring system. IEEE Sensors J..

[3] Castillejo P, Martínez JF, Rodríguez-Molina J, Cuerva A (2013). Integration of wearable devices in a wireless sensor network for an E-health application. IEEE Wireless Commun..

[4] Aziz O, Robinovitch SN (2011). An analysis of the accuracy of wearable sensors for classifying the causes of falls in humans. IEEE Trans. Neural Syst. Rehabil. Eng..

[5] Ranhotigmage, C. Human activities and posture recognition: Innovative algorithm for highly accurate detection rate. http://mro.massey.ac.nz/handle/10179/4339 (Dept. Eng. Electron. Comput. Syst. Eng., M.S. thesis, Massey Univ., 2013).

[6] Shany T, Redmond SJ, Narayanan MR, Lovell NH (2012). Sensors based wearable systems for monitoring of human movement and falls. IEEE Sensors J..

[7] Avci, A., Bosch, S., Marin-Perianu, M., Marin-Perianu, R. & Havinga, P. Activity recognition using inertial sensing for healthcare, wellbeing and sports applications: A survey. in *Proceedings of the 23rd International Conference on Architecture of Computing Systems, Hannover, Germany, 22–25 February 2010*. 1–10 (2010).

[8] Preece SJ, Goulermas JY, Kenney LP, Howard D, Meijer K, Crompton R (2009). Activity identification using body-mounted sensors-a review of classification techniques. Physiol. Meas..

[9] Guidoux R, Duclos M, Fleury G, Lacomme P, Lamaudiere N, Maneng P, Paris L, Ren L, Rousset S (2014). A smartphone-driven methodology for estimating physical activities and energy expenditure in free living conditions. J. Biomed. Inform..

[10] Costa, A., Andrade, F. & Novais, P. *Privacy and Data Protection towards Elderly Healthcare.**Handbook of Research on ICTs for Human-Centered Healthcare and Social Care Services*. 330–346 (2013).

[11] Shoaib M, Bosch S, Incel OD, Scholten H, Havinga PJ (2015). A survey of online activity recognition using mobile phones. Sensors.

[12] Vishwakarma S, Agrawal A (2013). A survey on activity recognition and behavior understanding in video surveillance. Vis. Comput..

[13] Chen L, Hoey J, Nugent CD, Cook DJ, Yu Z (2012). Sensor-based activity recognition. IEEE Trans. Syst. Man. Cybern. C Appl. Rev..

[14] Castelvecchi D (2016). Can we open the black box of AI?. Nat. News.

[15] Preece, A., Harborne, D., Braines, D., Tomsett, R. & Chakraborty, S. *Stakeholders in Explainable AI* (2018). arXiv:1810.00184.

[16] Gunning, D. Explainable artificial intelligence (xAI). in *Technical Reports* (Defense Advanced Research Projects Agency (DARPA), 2017).

[17] Tjoa, E., & Guan, C. *A Survey on Explainable Artificial Intelligence (XAI): Towards Medical XAI* (2019). arXiv:1907.07374. 10.1109/TNNLS.2020.3027314 33079674

[18] Zhu, J., Liapis, A., Risi, S., Bidarra, R., & Youngblood, G. M. Explainable AI for designers: A humancentered perspective on mixed-initiative co-creation. in *2018 IEEE Conference on Computational Intelligence and Games (CIG)*. 1–8 (2018).

[19] Uddin MZ, Hassan M, Alsanad A, Savaglio C (2020). A body sensor data fusion and deep recurrent neural network-based behavior recognition approach for robust healthcare. Inf. Fus..

[20] Kiranyaz S, Ince T, Gabbouj M (2016). Real-time patient-specific ECG classification by 1-D convolutional neural networks. IEEE Trans. Biomed. Eng..

[21] Hinton GE, Osindero S, Teh Y-W (2006). A fast learning algorithm for deep belief nets. Neural Comput..

[22] Deboeverie, F., Roegiers, S., Allebosch, G., Veelaert, P. & Philips, W. Human gesture classification by brute-force machine learning for exergaming in physiotherapy. in *Proceedings of IEEE Conference on Computational Intelligence and Games (CIG), Santorini*. 1–7 (2016).

[23] Graves, A., Mohamed, A., & Hinton, G. Speech recognition with deep recurrent neural networks. in *2013 IEEE International Conference on Acoustics, Speech and Signal Processing (ICASSP)*. 6645–6649. (IEEE, 2013).

[24] Hochreiter S, Schmidhuber J (1997). Long short-term memory. Neural Comput..

[25] Zaremba, W., Sutskever, I., & Vinyals, O. *Recurrent Neural Network Regularization*. arXiv preprint arXiv:1409.2329 (2014).

[26] Gers FA, Schraudolph NN, Schmidhuber J (2003). Learning precise timing with LSTM recurrent networks. J. Mach. Learn. Res..

[27] Sak H, Senior AW, Beaufays F (2014). Long short-term memory recurrent neural network architectures for large scale acoustic modeling. INTERSPEECH.

[28] Williams RJ, Peng J (1990). An efficient gradient-based algorithm for on-line training of recurrent network trajectories. Neural Comput..

[29] *Neural Structured Learning: Training with Structured Signals. Tensorflow. (Online)*. https://www.tensorflow.org/neural_structured_learning/. Accessed 01 Feb 2021 (2021).

[30] Bui, T. D., Ravi, S. & Ramavajjala, V. Neural graph learning. in *Proceedings of the Eleventh ACM International Conference on Web Search and Data Mining-WSDM ’18* (2018).

[31] Aghdam, H. H., Heravi, E. J. & Puig, D. Explaining adversarial examples by local properties of convolutional neural networks. in *Proceedings of the 12th International Joint Conference on Computer Vision, Imaging and Computer Graphics Theory and Applications* (2017).

[32] Tjoa, E. & Guan, C. A survey on explainable artificial intelligence (XAI): Toward medical XAI. in *IEEE Transactions on Neural Networks and Learning Systems*. 10.1109/TNNLS.2020.3027314. 10.1109/TNNLS.2020.3027314 33079674

[33] Mishra, S., Sturm, B. L. & Dixon, S. Local interpretable model-agnostic explanations for music content analysis. in *ISMIR*. 537–543 (2017).

[34] Ribeiro, M. T., Singh, S., & Guestrin, C. Nothing else matters: Model-agnostic explanations by identifying prediction invariance (2016). arXiv:1611.05817.

[35] Ribeiro, M. T., Singh, S., & Guestrin, C. Why should I trust you?: Explaining the predictions of any classifier. in *ACM SIGKDD International Conference on Knowledge Discovery and Data Mining*. 1135–1144 (ACM, 2016).

[36] Banos, O., Garcia, R., Holgado, J. A., Damas, M., Pomares, H., Rojas, I., Saez, A., Villalonga, C. mHealthDroid: A novel framework for agile development of mobile health applications. in *Proceedings of the 6th International Work-conference on Ambient Assisted Living an Active Ageing (IWAAL 2014), Belfast, December 2–5* (2014).

[37] Banos O, Villalonga C, Garcia R, Saez A, Damas M, Holgado JA, Lee S, Pomares H, Rojas I (2015). Design, implementation and validation of a novel open framework for agile development of mobile health applications. BioMed. Eng Online.

[38] Burns A, Greene BR, McGrath MJ, O’Shea TJ, Kuris B, Ayer SM, Stroiescu F, Cionca V (2010). Shimmer: A wireless sensor platform for noninvasive biomedical research. IEEE Sensors J..

[39] Kutlay MA, Gagula-Palalic S (2016). Application of machine learning in healthcare: Analysis on MHEALTH dataset. Southeast Eur. J. Soft Comput..

[40] Murdoch, W. J., Singh, C., Kumbier, K., Abbasi-Asl, R. & Yu, B. *Interpretable Machine Learning: Definitions, Methods, and Applications* (2019). arXiv:1901.04592. 10.1073/pnas.1900654116 PMC6825274 31619572

[41] Chander, A., Srinivasan, R., Chelian, S., Wang, J. & Uchino, K.Working with beliefs: AI transparency in the enterprise. in *Workshops of the ACM Conference on Intelligent User Interfaces* (2018).

[42] Chouldechova A (2017). Fair prediction with disparate impact: A study of bias in recidivism prediction instruments. Big Data.

[43] Kim, M., Reingold, O., & Rothblum, G. Fairness through computationally-bounded awareness. in *Advances in Neural Information Processing Systems*. 4842–4852 (2018).

[44] Tan, S., Caruana, R., Hooker, G. & Lou, Y.Distill-and-compare: Auditing black-box models using transparent model distillation. in *AAAI/ACM Conference on AI, Ethics, and Society*. 303–310 (ACM, 2018).

[45] Gajane, P. & Pechenizkiy, M. *On Formalizing Fairness in Prediction with Machine Learning* (2017). arXiv:1710.03184.

[46] Dwork, C. & Ilvento, C. *Composition of Fairsystems* (2018). arXiv:1806.06122.

[47] Barocas, S., Hardt, M. & Narayanan, A. *Fairness and Machine Learning, fairmlbook.org*. http://www.fairmlbook.org (2019).

[48] Burns, K., Hendricks, L. A., Saenko, K., Darrell, T., & Rohrbach, A. *Women also Snowboard: Overcoming Bias in Captioning Models* (2018). arXiv:1803.09797.

[49] Bennetot, A., Laurent, J.-L., Chatila, R. & D´ıaz-Rodr´ıguez, N. Towards explainable neural-symbolic visual reasoning, in *NeSy Workshop IJCAI 2019, Macau, China* (2019).

[50] Palumbo F, Gallicchio C, Pucci R, Micheli A (2016). Human activity recognition using multisensor data fusion based on reservoir computing. J. Ambient Intell. Smart Environ..

